# On the functional independence of numerical acuity and visual working memory

**DOI:** 10.3389/fpsyg.2024.1335857

**Published:** 2024-03-13

**Authors:** Roberto Dell’Acqua, Paola Sessa, Sabrina Brigadoi, Judit Gervain, Roy Luria, Mattia Doro

**Affiliations:** ^1^Department of Developmental Psychology, University of Padova, Padua, Italy; ^2^Padova Neuroscience Center, University of Padova, Padua, Italy; ^3^The School of Psychological Sciences, Tel Aviv University, Tel Aviv, Israel; ^4^Sagol School of Neuroscience, Tel Aviv University, Tel Aviv, Israel

**Keywords:** visual working memory, approximate number system, correlation, attention, sensory system acuity

## Abstract

Deciding where to direct our vehicle in a crowded parking area or where to line up at an airport gateway relies on our ability to appraise the numerosity of multitudes at a glimpse and react accordingly. Approximating numerosities without actually counting is an ontogenetically and phylogenetically primordial ability, given its presence in human infants shortly after birth, and in primate and non-primate animal species. Prior research in the field suggested that numerosity approximation is a ballistic automatism that has little to do with human cognition as commonly intended. Here, we measured visual working memory capacity using a state-of-the-art change detection task and numerosity approximation using a dot-comparison task, and found a null correlation between these two parametrical domains. By checking the evidential strength of the tested correlation using both classic and Bayesian analytical approaches, as well as the construct validity for working memory capacity and numerosity approximation estimates, we concluded that the present psychophysical evidence was sufficiently strong to support the view that visual working memory and numerosity approximation are likely to rely on functionally independent stages of processing of the human cognitive architecture.

## 1 Introduction

Working memory is a pivotal construct in large-scale models of human cognition (e.g., Meyer and Kieras, 1997; Dehaene et al., 1998; Anderson, 2007), in which it is incorporated as a core workspace for the active maintenance of information gathered through our senses (Jolicœur and Dell’Acqua, 1998), or retrieved from long-term memory (Fukuda and Woodman, 2017). The capacity of working memory is starkly limited, amounting to 3 objects on average in the human adult population, and characterized by a substantial inter-individual variability (Cowan, 2001; Luck and Vogel, 2013; Balaban et al., 2019). Although the functional characterization of such limits is still debated (*cf.*, Bays and Husain, 2008; Zhang and Luck, 2008; see, however, Pratte and Green, 2023), studies employing electroencephalographic recordings have provided empirical evidence that working memory capacity is inextricably intertwined with the ability to protect working memory from clutter or distraction, showing that individuals with high working memory capacity are also particularly efficient in using that capacity to store task-relevant rather than irrelevant information (Vogel et al., 2005; Gaspar et al., 2016).

Working memory capacity is predictive of such an impressively wide range of measures of cognitive efficiency tapping basically all high-level psychological constructs (Miyake et al., 2001; Cowan et al., 2005; Johnson et al., 2013; Unsworth et al., 2014, 2015) that one issue worthy of investigation is uncovering aspects of human behavior that are not influenced by working memory capacity. Focusing on this issue has the potential to deepen our general understanding of which stages of processing within a human cognitive architecture can take place independently of working memory and, concomitantly, to model more precisely the influence of working memory on human mental life.

One candidate ability in this perspective is that of approximating the numerosity of objects in a bunch when this number is large. Choosing a parking spot in a crowded parking area or the line of people at an airport gateway are everyday examples where this ability is typically exerted, often by simply glimpsing at the situation and reacting accordingly. This ability is usually referred to as numerical acuity, and defined as the efficiency to categorize as different the cardinality of sets of objects varying in ratio. In essence, the higher a person’s numerical acuity, the smaller the numerical difference between two sets of objects the person needs in order to categorize them as different, without resorting to verbal and/or mental count (Gallistel and Gelman, 1992; Dehaene, 2011). Studies in developmental psychology and animal cognition suggest number approximation is phylogenetically and ontogenetically ancestral. We share this ability with a number of primate and non-primate animal species, and it is present in human infants a few months after birth, following a developmental trajectory that is largely independent of that of other higher-level abilities, such as language (Barth et al., 2003; Nieder and Dehaene, 2009).

Hints that numerosity approximation is indeed functionally separable from working memory have been reported by Piazza et al. (2011), in a study in which working memory capacity was estimated using a whole-display change detection task, in which participants had to compare memory and probe arrays of color patches in order to detect a possible change in one color. Visual working memory capacity was calculated as K, an index of the number of working memory ‘slots’ available for storing distinct objects (Cowan, 2001). Numerosity processing was explored both as the ability to ‘subitize’ a small number of dots in a visual array (e.g., Trick and Pylyshyn, 1994), and as the ability to discriminate two concomitant visual arrays of dots based on their ‘approximated’ (i.e., estimated without relying on explicit counting; e.g., Piazza et al., 2004) numerosity. Subitizing capacity was measured in a dot counting task, in which 1 to 8 colored dots were displayed for 250 ms and participants were instructed to name the number of the dots as fast as they could. Subitizing capacity was estimated as the average number of dots after which naming times began to increase (Revkin et al., 2008). Numerosity approximation, by contrast, was tested in a dot-comparison task, in which up to 44 dots were divided into two arrays of unequal number (with large/small numerosity ratios of 1.06, 1.14, 1.23, 1.33, or 1.6) and displayed to the left and right of fixation until participants pressed one of two buttons to indicate the array with a larger dot numerosity. Number approximation ability, also known as numerical acuity, was calculated based on accuracy in the dot-comparison task and expressed as a Weber fraction reflecting the degree of resolution in distinguishing quantities varying in ratio (Dehaene, 1993). The results showed that working memory capacity was positively correlated with subitizing capacity, and not correlated with numerical acuity. Converging with earlier analogous proposals (e.g., Drew and Vogel, 2008; see also Ester et al., 2012), Piazza et al. (2011) concluded that subitizing capacity relies on a multi-purpose, supra-modal (Gennari et al., 2023), attention-demanding system representing cardinalities as a limited number of discrete entities encoded in visual working memory, whereas numerical acuity relies on a primordial, likely pre-attentive (Burr et al., 2010), system for magnitude estimation that does not use up visual working memory resources.

Though favorably inclined to believe that the absence of a correlation between numerical acuity and visual working memory capacity shown by Piazza et al. (2011) is real, we noted important issues pertaining to both the design of the change detection task and the way in which *K* was calculated by Piazza et al. (2011) that motivate the present attempt at providing a stronger test for a possible dissociation between visual working memory and numerical acuity. In the change detection task used by Piazza et al. (2011), for instance, the memory array was displayed for 700 ms and followed by a blank 1,000 ms inter-stimulus interval (ISI) prior to the onset of the test array, which was exposed for 2000 ms. This is a significant deviation from designs typically employed to estimate visual working memory capacity, where the timing parameters are often chosen so as to prevent participants from adopting idiosyncratic strategies for task execution. The memory array, for instance, is typically exposed for a much briefer duration, or better, so brief as to prevent participants from directing their foveae to a subset of the to-be-memorized objects rather than maintaining gaze at fixation. By keeping the exposure duration short enough to prevent eye movements and by using a blank ISI of a maximum of 1,000 ms, other strategies can also be precluded, like, for instance, the phonological recoding of the memoranda (Ramaty and Luria, 2018), i.e., encoding and rehearsing the names of the colors composing the memory array, which is hypothesized to implicate a hand-off of part of the visual working memory content to long-term memory and/or the engagement of verbal circuits (Luria et al., 2010). Such strategies could mask real individual differences in VWM capacity and dilute any correlation with another variable.

Over and above these issues in the design, we found the way in which Piazza et al. (2011) estimated and used *K* for correlation testing even more troublesome. As described by Rouder et al. (2011), on the assumption that visual working memory resources are fixed and discretely subdivided among a limited number of memorized colored stimuli, the formula to be used for the calculation of *K* when using whole-display change detection tasks must be the one proposed by Pashler (1988), and not that proposed by Cowan (2001). Pashler’s (1988) formula takes into account the probability to detect a change in any unpredictable color composing a whole-display probe array, whereas Cowan’s (2001) formula only applies to conditions in which a single item is used to probe working memory for a color that occupied a specific position in the memory array. Despite the use of a whole-display change detection task, Piazza et al. (2011) used Cowan’s (2001) formula. Symptomatic of this arguable choice was the maximum *K* found when 8 colors had to be memorized, which was greater than *K* when 6 or 7 colors had to be memorized, indicating that *K* did not plateau as expected, and that this was likely one of the cases that Rouder et al. (2011) levied to argue against an interchangeable use of Pashler’s (1988) and Cowan’s (2001) formulae. Furthermore, if the correlation test sought to explore whether the upper limits of visual working memory impose constraints on numerical acuity, one should consider the maximum *K* values observed when the memory array’s set-size is supra-capacity, and not, as Piazza et al. (2011) did, the average *K* value across the whole range of tested set-sizes, which varied from 1 to 8 colors. At set-sizes inferior or equal to visual working memory capacity (i.e., set-sizes 1–3), the maximum *K* is constrained by set-size, and the average *K* is therefore an underestimation of the maximum *K* (Balaban et al., 2019).

As illustrated in the forthcoming sections, we designed the present experiment to test a possible correlation between individual maximum *K*s and numerical acuity, in an attempt to overcome the above-described limitations of the test carried out by Piazza et al. (2011). To anticipate, after overcoming these limitations, the results were reassuring, for we detected no correlation, thus providing solid evidence for the independence of these two abilities and strengthening considerably the view that numerical acuity and visual working memory are indeed likely to rely on functionally independent stages of processing in the human cognitive architecture, whereby working memory is a high-level central hub for information processing and numerosity approximation is enabled by lower-level, likely sensory, processing stages.

## 2 Methods

### 2.1 Participants

Given the expected result was a nil correlation between estimates of numerical acuity and visual working memory, a standard power analysis — which is intended to determine the number of participants required to attain a pre-determined statistical power to detect a significant effect — would have resulted in an abnormally large number of participants (in the order of hundreds). To circumvent this problem, our approach was that of hypothesizing that a correlation could indeed be found, with Pearson’s *r*s ranging from 0.4 and 0.6. To detect such effects, the number of participants required to attain a statistical power of 0.8 and an alpha of 0.05 ranged from 18 to 45. Forty-six students from the local University (27 women, 19 men) were thus recruited to take part in the present experiment after giving informed consent. All participants reported normal or corrected-to-normal vision and no history of neurological and/or psychiatric disorders. Their mean age was 22.7 years (SD = 2.7). Three participants were removed due to less than 60% correct responses in the change detection task, and two participants were removed because of mean performance values (see below) exceeding the sample mean by three standard deviations in the dot-comparison task. The final sample included 41 participants (23 female, 18 male, mean age = 22.9, SD = 2.7). The experimental protocol was vetted by the local Ethical Committee (Protocol #4683).

### 2.2 Stimuli and procedure

The stimuli of the change detection and dot-comparison tasks were generated with E-Prime 2 software (Psychology Software Tools Inc.) and MATLAB (Version: 9.13.0, R2022b, The MathWorks Inc.), respectively, and displayed on the black (RGB: 0, 0, 0) background of a 24” CRT monitor with a refresh rate of 60 Hz at a distance of about 65 cm. The two tasks were administered on the same day, one after the other following a pause, in counterbalanced order across participants.

**Figure 1:**
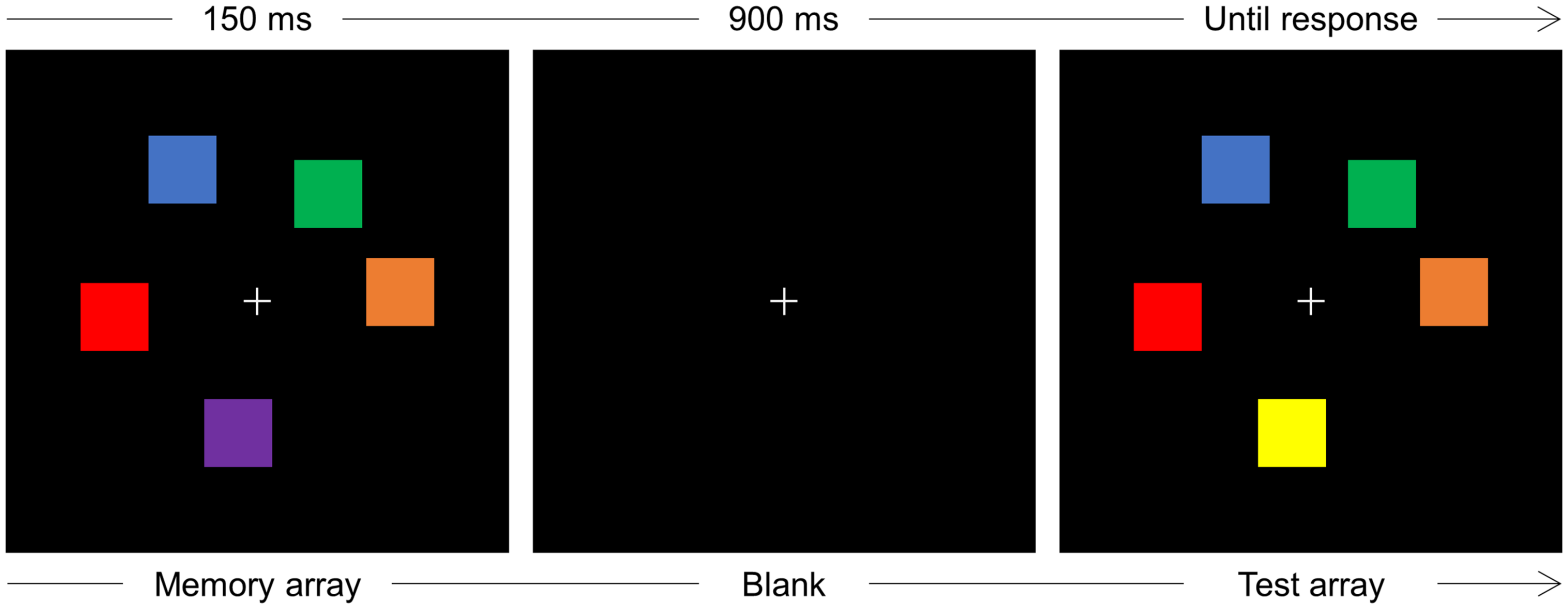
Schematic illustration of the sequence of events on one trial of the change detection task. Participants were instructed to maintain gaze at fixation (+) and memorize the colors of a varying number (2–5) of squares (memory array) displayed for 150 ms. After a blank interval of 900 ms, a test array was displayed that could be identical to the memory array or, as is the case illustrated in the figure, a change in color occurred for one square. The test array remained in view until response detection.

An example of the stimuli and a schematic illustration of the sequence of events in the change detection task is reported in Figure 1.

Each trial started when the participant pressed the spacebar of the keyboard positioned in front of them. Upon spacebar press, a light gray (RGB: 220, 220, 220) fixation cross, subtending 0.8 × 0.8° of visual angle, was displayed at the center of the screen for a 900–1,000 ms interval, randomly jittered in steps of 20 ms. A memory array composed of two, three, or five colored squares was then displayed around fixation for 150 ms. Each square subtended 1° × 1° and the colors were randomly chosen among blue (RGB: 0, 0, 255), brown (RGB: 157, 0, 23), orange (RGB: 255, 128, 0), purple (RGB: 128, 0, 255), dark green (RGB: 30, 140, 60), cyan (RGB: 0, 255, 255), pink (RGB: 255, 174, 201), magenta (RGB: 255, 0, 255), yellow (RGB: 255, 255, 0), red (RGB: 255, 0, 0), and light green (RGB: 0, 255, 0). The colored squares could be displayed at random positions within a notional rectangle subtending 5° × 5°, within the constraints that the minimum distance between the upper left corners of two adjacent squares could be no less than 1.5°, and the minimum distance between the fixation point and the side of the nearest square could be no less than 1.3°. After a 900 ms blank retention interval, a test array was displayed that was, with equal probability, identical to the test array or different from the memory array for the change in one color. The test array remained in view until participants pressed one of two keys (i.e., the keys “1” or “2” of the numeric keypad, counterbalanced across participants) to indicate whether memory and test arrays were the “same” or “different” for one color. The experiment was composed of 810 trials, organized in 27 blocks of 30 trials each, and preceded by 16 trials of practice.

**Figure 2:**
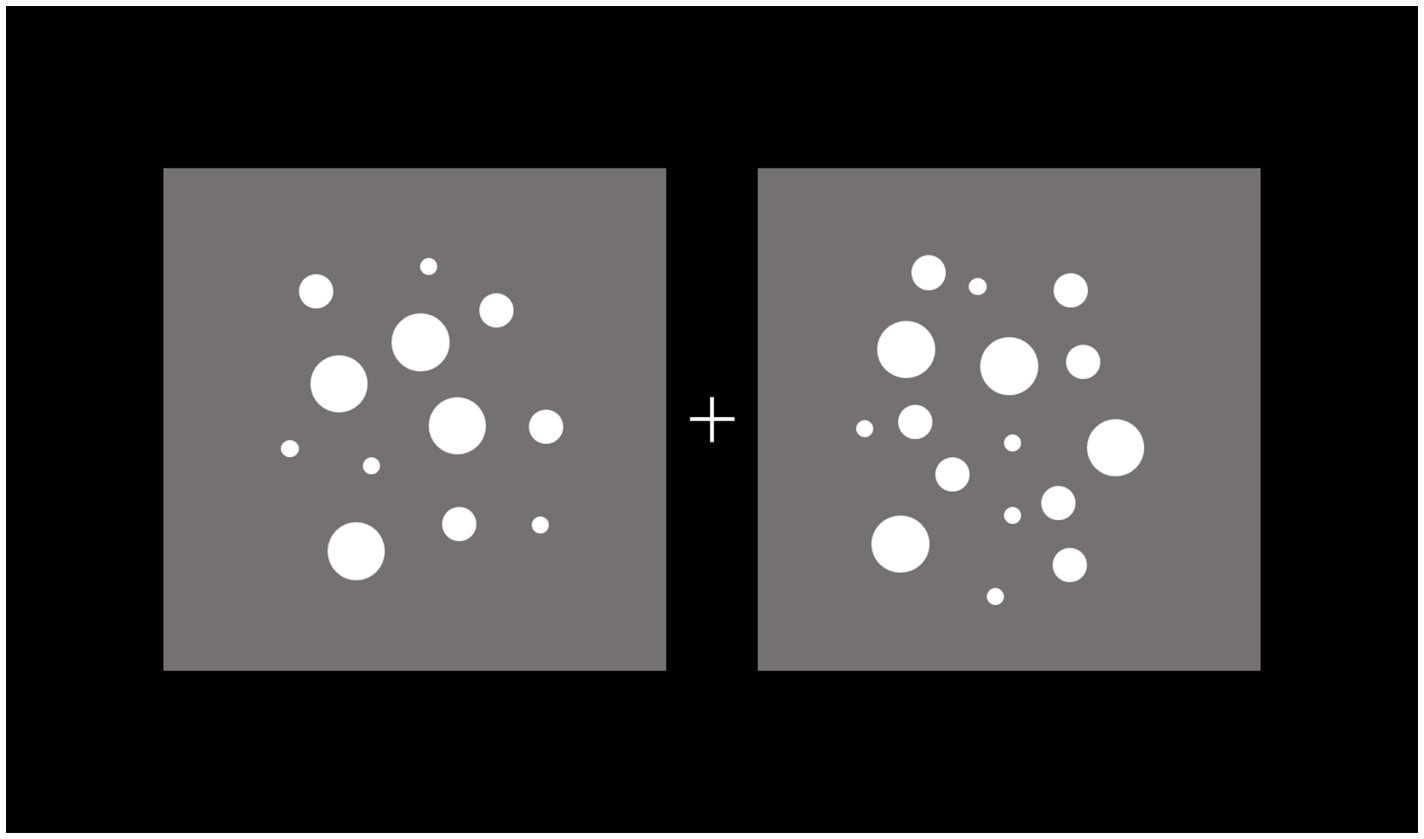
Example of the stimuli in the dot-comparison task. Participants were instructed to maintain gaze at fixation (+) and press, within a maximum interval of 2 s, one of two buttons to indicate the side (left vs. right) of the dots array of larger numerosity. The stimuli remained in view until response detection.

An example of the stimuli used in the dot-comparison task is reported in Figure 2.

Two arrays composed of white (RGB: 255, 255, 255) dots were separately included in two gray (RGB: 128, 128, 128) square areas positioned to the left and right of a central light gray (RGB: 220, 220, 220) fixation cross subtending 0.8 × 0.8°.

Each trial consisted of two arrays of dots, in which one array served as reference with a numerosity constrained to be of either 16 or 32 dots. The numerosity of the other array was systematically varied according to the numerosity of the reference array. If the reference array contained 16 dots, the numerosity of the other array ranged, with equal probability, from 12 to 20 dots. If the reference array contained 32 dots, the numerosity of the other array ranged, with equal probability, from 24 to 40 dots. This experimental design resulted in 8 distinct tested large/small numerosity ratios, that is, 1.063, 1.067, 1.125, 1.143, 1.188, 1.231, 1.250, and 1.333. To counterbalance the influence of physical/sensory factors covarying with numerosity, we used the Matlab toolbox CUSTOM (De Marco and Cutini, 2020), such that both arrays had equal dot density in a random 50% of trials, and covered an equal total surface area in the other 50% of trials.

An important methodological note is in order about the use of this specific software for stimuli generation. As is well-known, a number of continuous variables naturally covary with numerosity when expressed non-symbolically. For instance, a larger amount of dots occupies a larger space area, has a greater total surface and a greater convex hull, such that one might argue — as done by some in the literature (e.g., Leibovich et al., 2017) — that a dot-comparison task does not tap into number processing, but into the detection of some combination of continuous variables. However, the software CUSTOM (De Marco and Cutini, 2020) provides a state-of-the-art solution for decorrelating numerosity and perceptual variables. Density control is a sophisticated aspect of the algorithm, as it goes beyond traditional density measures by introducing an innovative metric based on inter-item spacing. The algorithm’s incorporation of randomness implies that when the same input parameters are used, all generated images will exhibit precisely identical visual features, despite appearing visually different from one another. The constraints implemented in CUSTOM are of geometrical nature, indicating that the algorithm is completely unbound by any theoretical perspective. In addition, to further minimize the chance of the presence of either sensory or response biases induced by our stimuli, we generated a set of 1,280 pairs of arrays of dots for each participant and randomly sampled 192 pairs of dot arrays that were displayed on separate experimental trial, organized in 12 blocks of 16 trials each.

Participants were instructed to indicate the side (“left” vs. “right”) of the array of larger numerosity by pressing a spatially corresponding key of the computer keyboard (“A” for left and “L” for right). The arrays remained on the screen until participants made their response within a maximum interval of 2 s. A short practice phase composed of 16 trials preceded the experimental phase.

## 3 Results

### 3.1 Change detection task

Mean proportions of correct responses were 0.95 for set-size 2, 0.91 for set-size 3, and 0.80 for set-size 5. Individual accuracy proportions were submitted to an ANOVA with set-size (2, 3, and 5) as a within-participant factor. The results revealed a significant difference in accuracy across set-sizes [*F*(2,80) = 209.4, 𝜂^2^_p_ = 0.840, *p* < 0.001]. *Post-hoc* comparisons were conducted via three independent *t*-tests using the false discovery rate (FDR; Benjamini and Hochberg, 1995) correction for multiple comparisons. All *t*-tests yielded significant results, reflecting the fact that accuracy for set-size 2 (0.95) was higher relative to accuracy at set-size 3 [0.91; *t*(40) = 9.3, *p* < 0.001], and accuracy at set-size 5 [0.80, *t*(40) = 14.3, *p* < 0.001], with these two latter accuracy values also differing from each other [*t*(40) = 15.5, *p* < 0.001].

The individual average number of colors memorized at each set-size was estimated in terms of K, which was calculated using Pashler’s (1988) formula:


$$\hat{K}=N\left(\frac{\hat{h}-\hat{f}}{1-\hat{f}}\right)$$

where $\hat{h}$ and $\hat{f}$ are the observed hit and false alarm proportions, respectively, and N is the set-size.

Individual *K* values were submitted to an ANOVA with set-size as a within-participant factor. The results revealed a significant difference in memory capacity across set-sizes [*F*(2, 80) = 128.6, 𝜂^2^_p_ = 0.974, *p* < 0.001]. *Post-hoc* comparisons were conducted via three independent FDR-corrected *t*-tests. All *t*-tests yielded significant results, reflecting the fact that *K* at set-size 2 (1.9) was smaller relative to both *K* at set-size 3 [2.8; *t*(40) = −42.4, *p* < 0.001], and *K* at set-size 5 [4.4, *t*(40) = −31.4, *p* < 0.001], with these two latter *K* values differing from each other [*t*(40) = −42.9, *p* < 0.001].

**Figure 3:**
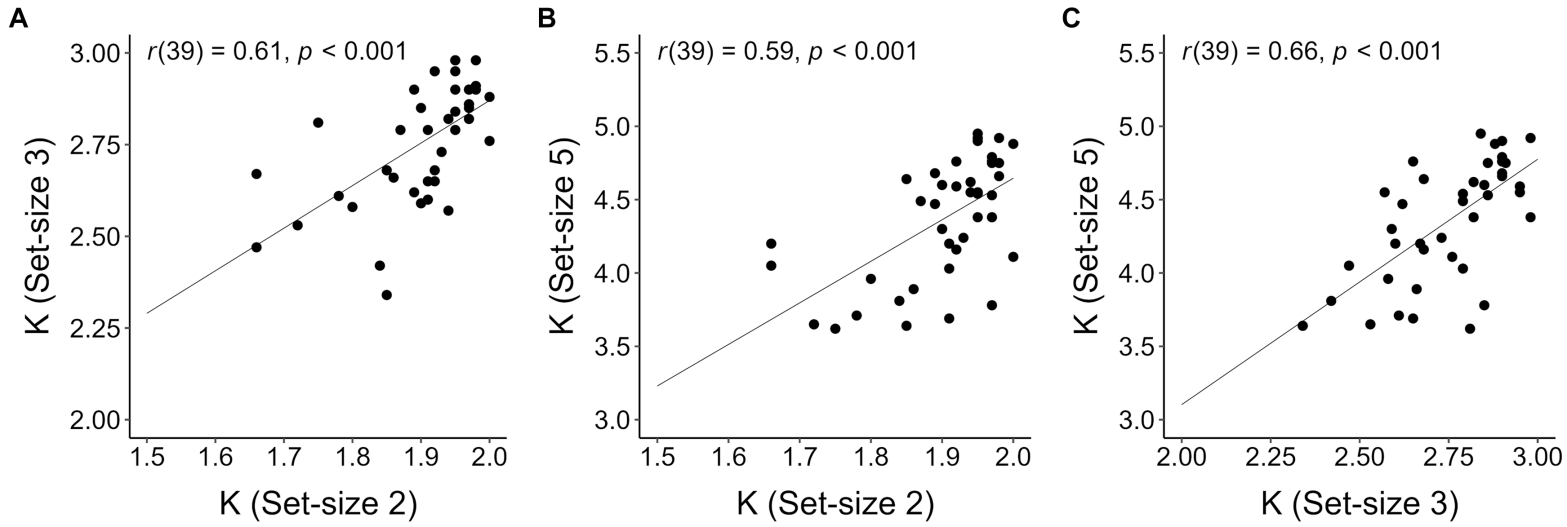
Correlations between individual Ks computed for different set-sizes. **(A)** Set-size 2 and set-size 3; **(B)** set-size 2 and set-size 5; **(C)** set-size 3 and set-size 5. The lines on each plot represent the estimated regression lines.

To inspect whether our data met the criterion for *K* construct validity proposed by Balaban et al. (2019), we checked whether *K* correlated across each pair of set-sizes. Balaban et al. (2019) argument for this check was that if *K* at each set-size validly estimates the number of stored colors in visual working memory, then *K* at each set-size should be predictive of (i.e., correlate with) *K* at each other set-size. As is apparent in Figure 3, *K* values in the present context met this criterion (Pearson’s *r*s reported in the insets).

Additionally, we estimated d’ (Green and Swets, 1974) to inspect the memory strength (i.e., signal quality and discriminability) of visual working memory representations. *d*’ values reflect the individual capacity to differentiate between remembered and non-remembered items, thereby capturing the ability to distinguish between these two states of visual working memory representations. To calculate d’, we used the following formula:


$$d^{\prime }=Z\left(\hat{h}\right)-Z\left(\hat{f}\right)$$

where $Z\left(\hat{h}\right)$ and $Z\left(\hat{f}\right)$ are the Z-score of the hit rate and of the false alarm rate, respectively. As in Balaban et al. (2019), *d*’ decreased as the set-size increased (*d*’ at set-size 2 = 3.53; *d*’ at set-size 3 = 2.85; *d*’ at set-size 5 = 2.01), indicating a progressive decrease in memory signal strength as set-size increased.

**Figure 4:**
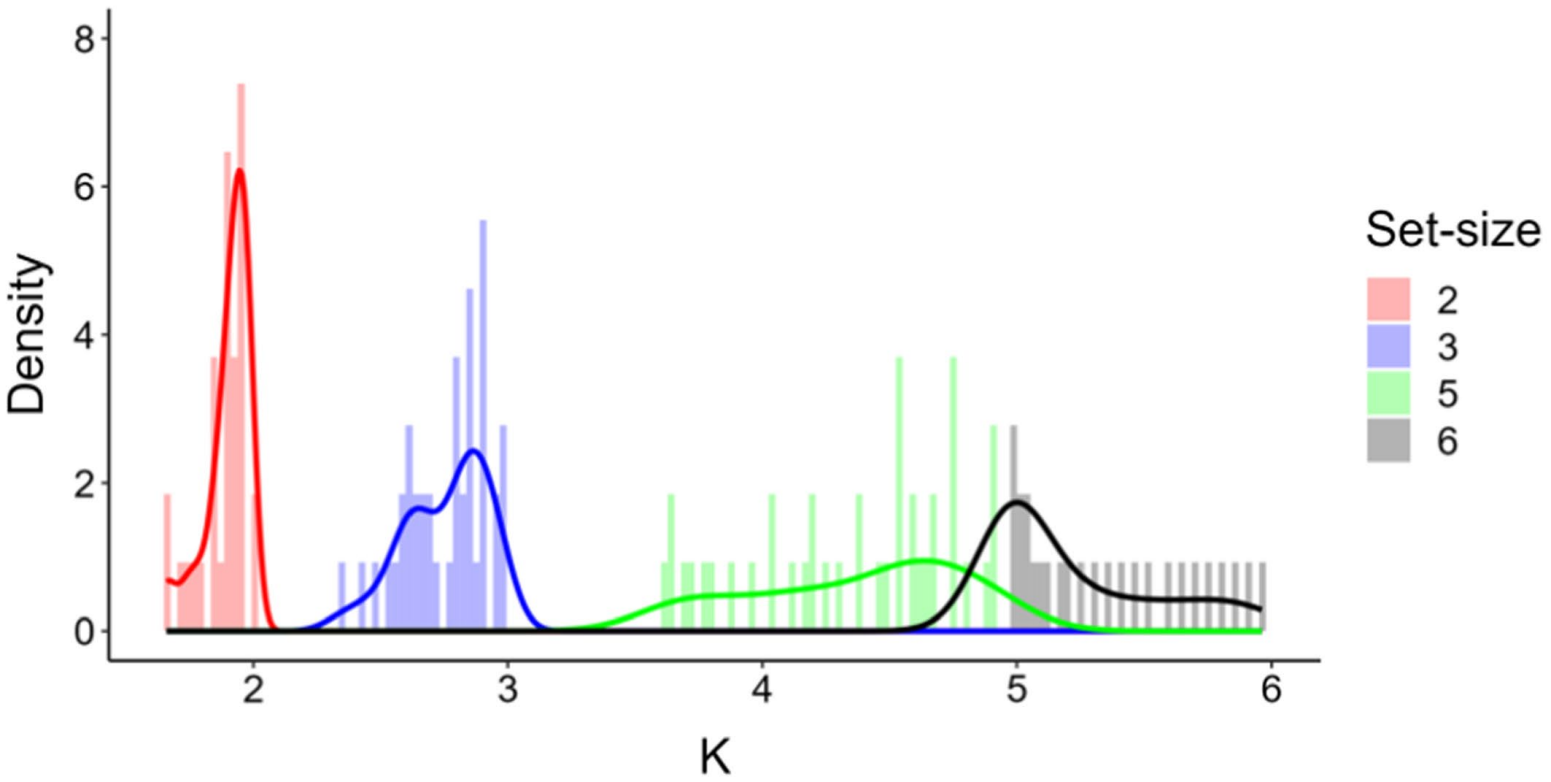
Frequency distributions of real (red, blue, and green, set-size 2, 3, 5, respectively) and simulated *K* values (black, set-size 6).

The progressive increase of mean *K*s as set-size was increased could suggest that *K* values at set-size 5 did not reflect the expected plateau of maximum memory capacity for all participants. To check for this possibility, we simulated *K* values for an artificial set-size 6, and inspected whether this simulated *K* would exceed the real *K* value observed at set-size 5. In order to predict *K* at set-size 6, we fitted four linear polynomials to a subset of moments of the distribution of *K* values observed at set-sizes 2, 3, and 5, namely, mean (1.9, 2.8, and 4.4, respectively), standard deviation (0.09, 0.16 and 0.41, respectively), skewness (−1.41, −0.81, and −0.42, respectively), and kurtosis (4.1, 3.0, and 1.9, respectively). Based on these values, we generated 10,000 random distributions of 41 (equal to the participants’ numerosity) artificial *K* values using the function ‘rpearson’ of the PearsonDS (Becker and Klößner, 2023) package in R (R Core Team), after constraining the range of possible set-sizes from 0 to 6. The density plots of the real and simulated Ks (averaged across 10,000 artificial values) for each set-size are shown in Figure 4. The simulated data for set-size 6 showed that a minority of participants did not in fact reach their individual maximum memory capacity at set-size 5. Importantly, however, the density plot for set-size 6 is clear in indicating that, in the present artificially generated scenario, most participants would have reported a *K* value peaking around a median *K* value of 5.03, implying that at least half of the participants had reached their maximum visual working memory capacity at set-size 5.

### 3.2 Dot-comparison task

Individual Weber fractions (ω) were calculated as an index of each participant’s numerical acuity. The computation of this parameter was based on the proportion of correct responses at each numerosity ratio, using the formula proposed by Piazza et al. (2004):


$$P_{larger}\left(N,\;Nref\right)=\frac{1}{2}\;\left(1+erf\left(\frac{\log\left(\frac{N}{Nref}\right)}{\sqrt{2}\;\omega }\right)\right)$$

where *P*_larger_ is the probability of responding larger on a trial on which a reference dot array of Nref numerosity (varying between 16 and 32) had to be compared with a dot array of N numerosity, and erf is the error function.

Overall, participants showed an average ω value of 0.20 (SD = 0.07, data fit: *R*^2^ = 0.81, SD = 0.14). Given a smaller ω indicates greater acuity in discriminating between dot numerosities, we reversed the (somewhat counterintuitive) relationship by analyzing and plotting numerical acuity values as 1 – ω, such that a greater 1 – ω value indicates greater acuity. Additionally, we independently estimated ω for trials on which the reference frame included 16 dots and on which the reference frame included 32 dots, finding ω values that did not differ from ω computed considering all trials (mean = 0.22, SD = 0.09; mean = 0.19, SD = 0.08, respectively), resulting also from comparable fits (*R*^2^ = 0.85, SD = 0.12; *R*^2^ = 0.85, SD = 0.11, respectively). To evaluate the reliability of ω, we correlated individual ω between trials varying in reference frame dot numerosity (16 vs. 32) finding a significant result [*r*(39) = 0.67, *p* < 0.001].

### 3.3 Correlation between visual working memory capacity and numerical acuity

**Figure 5:**
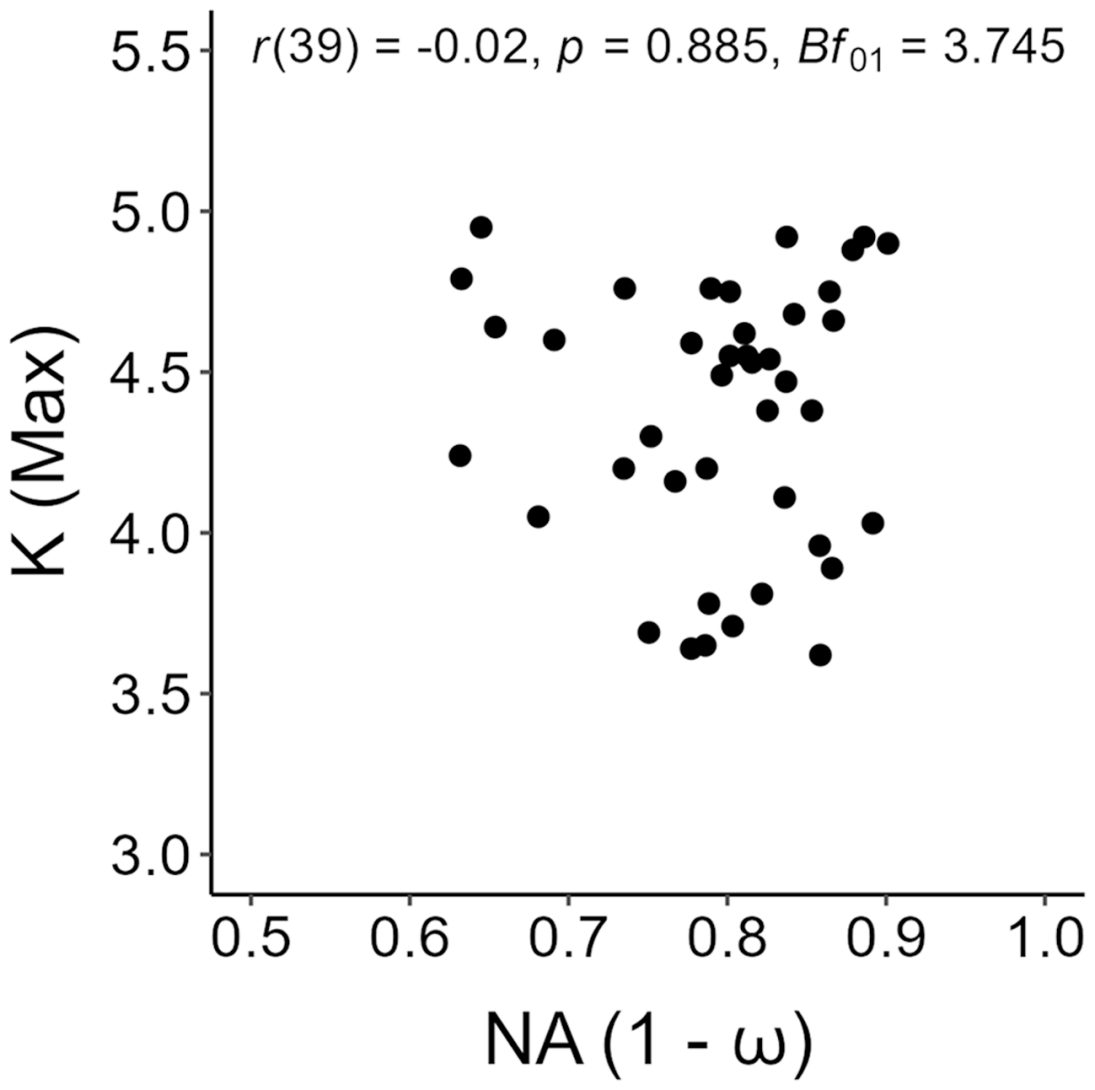
Scatterplot of individual numerical acuity (NA) values in the dot-comparison task and maximum *K* values in the change detection task. Pearson’s *r* and the Bayes factor of the correlation analysis are reported in the inset.

We conducted a correlation analysis between individual values of numerical acuity and the maximum *K* reached at set-size 5, after ascertaining no participant reached a maximum *K* value at smaller set-sizes. Given the expected null result based on the work of Piazza et al. (2011), we computed the Bayes factor (Bf_01_) using a beta distribution as prior with $1/\sqrt{3}$ scale so as to substantiate an expected higher likelihood of the null hypothesis (i.e., absence of a correlation between visual working memory capacity and numerical acuity) against the alternative hypothesis (i.e., presence of a correlation between visual working memory capacity and numerical acuity). The choice of this prior distribution, often defined as “wide” (e.g., Ly et al., 2016), was made so as to allow us to consider every possible correlation value without constraining the analysis to a predetermined range of correlation values. The scatterplot with the values are shown in Figure 5, with Pearson’s *r* and the Bayes factor reported in the insets. Based on a standard approach to hypothesis testing, the results were clear-cut in showing absence of a correlation between visual working memory and numerical acuity. Furthermore, the Bayes factor supported these results, suggesting that the evidence in favor of the absence of a correlation for both the present tests was ‘substantial’ (Jeffreys, 1939).

## 4 Discussion

Our investigation aimed to establish evidence for the relationship between working memory capacity and numerical acuity. We implemented a particularly stringent test, eliminating possible confounds between numerosity and different analogical dimensions and preventing participants from using irrelevant strategies to solve the tasks. First, we used timing parameters in the design of the change detection task to discourage participants from foveating on individual items, engaging in verbal rehearsal, and/or employing idiosyncratic strategies. Second, we generated the dots in the numerical acuity task such that numerosity was not correlated with perceptual variables. Furthermore, we employed a more appropriate formula, as proposed by Pashler (1988), to calculate estimates of working memory capacity (i.e., *K*s). These methodological choices ensured that our measurement of working memory capacity was aligned with current theoretical frameworks and provided the most rigorous possible measure of participants’ capacity.

Importantly, we did not find a significant correlation between these psychophysical estimates of working memory capacity (K) and numerical acuity (ω). To bolster the statistical support for our null result, we conducted a Bayesian analysis. This analysis framework offers valuable insights for interpreting evidence in favor of the null hypothesis. Incorporating a Bayesian analysis in our study provides crucial insight. It acknowledges that a null result can be meaningful, serving as evidence not compatible with the presence of a correlation. By showing that the strength of the evidence favoring the null hypothesis is substantial, our study excludes alternative, theoretically less relevant, explanations for the null results such as lack of statistical power.

While we found a null correlation between working memory capacity and numerical acuity, it is important to note that psychophysical effects separately indexed by these distinct measures were successfully detected after double-checking their reliability. Values of numerical acuity were highly correlated across levels of absolute reference numerosity. *K* values were also highly correlated across memory array set-sizes. After checking for these measures’ reliability, we observed a significant difference in working memory capacity estimates among participants when memorizing an increasing number of items (i.e., 2, 3, or 5 items), in the form of a progressive increase in *K* values as the set-size increased. In addition, as the set-size increased, there was a decrease in *d*’ values, reflecting a progressive decline of the ability to reliably differentiate between remembered and non-remembered items. In the dot-comparison task, we assessed the precision of numerical approximation (i.e., numerical acuity) using the internal Weber fraction (ω). The average ω value of 0.20 exhibited by our participants indicates good numerical discrimination ability, reflecting a generally high acuity in discriminating between quantities. An unexpected result was observed when simulating *K* for a hypothetical set-size of 6. We did this in order to ascertain that *K* values at set-size 5 truly reflected the maximum working memory capacity for all participants, and that no further increase in *K* would be found at set-size 6, even for a subset of participants. However, the comparison between *K* values at set-sizes 5 and 6 revealed that, although the mean *K* at set-size 6 amounted to 5.03, some participants were not at capacity with set-size 5, implying that our change detection design was perhaps suboptimal to detect a memory plateau at the largest (not simulated) set-size. As a partial justification, we replicated the design of Fukuda et al. (2015), who tested 495 participants. These authors found that *K* values plateaued at 3 at set-size 4, with *K* eventually decreasing at larger set-sizes. In light of this result and the particularly large sample size in Fukuda et al.’s (2015) study, our choice of set-size 5 was aimed at determining a *K* value that reflected a memory plateau. Since Fukuda et al. (2015) study was based on a much larger sample size than ours, we deem it quite unlikely that a zero correlation between working memory capacity and numerical acuity at set-size 5 would have been obtained and become significant, had we used a set-size 6 (or larger).

The absence of a correlation between working memory capacity and numerical acuity holds important theoretical implications. It suggests that these cognitive processes rely on distinct mechanisms and, likely, distinct neural networks. The observed independence between psychophysical estimates of working memory capacity and numerical acuity may support the notion of a modular cognitive architecture, where specialized subsystems carry out different functions. It also raises the possibility of a hierarchical organization, with working memory being hierarchically superior in the human cognitive architecture to a lower-level numerical approximation ability that is likely to be a reflection of a sensory routine. This proposal is compatible with models like that of Burr and Ross (2008), based on a study showing that, as is typical for a range of physical signals in all sensory modalities, numerosity approximation is subject to habituation. Burr and Ross (2008) had subjects habituate for 30 s to two arrays of items of different numerosity displayed to the left and right of fixation, followed by two equinumerical arrays in the same position. Strikingly, subjects consistently underestimated the array displayed in the same position as the habituating array of larger numerosity and overestimated the other. The sensory nature of the visual signal that ultimately determines numerical acuity may also provide a novel perspective on the absence of a correlation between numerical acuity and visual working memory. Specifically, visual working memory is a multi-faceted construct in which multiple factors contribute to its known limitations. A plethora of studies focusing on an established ERP hallmark of information maintenance in visual working memory, i.e., the sustained posterior contralateral negativity (or SPCN; Lefebvre et al., 2011; also known as contralateral delay activity, or CDA; Luck and Vogel, 2013), have long converged on the idea that two factors are key in modulating visual working memory efficiency. One is capacity, broadly defined as the amount of space used to store information and maintain it in an active state. Typically, visual working memory capacity is held to be indexed by amplitude increments of SPCN activity (i.e., the net increment in negativity resulting from subtracting the ipsilateral activity detected at parietal electrodes from the contralateral equivalent) proportional to the set-size of a memory array in change detection tasks (Luria et al., 2010). Critically, however, SPCN amplitude variations also capture a second factor, namely, attention control efficiency. This is supported by studies showing that individuals with particularly high *K* values show SPCN amplitude increments when presented with information that is relevant for the task at hand, but not when they are presented with irrelevant, and potentially distracting, information. In striking contrast, individuals with low *K* values, besides a generally attenuated SPCN response to memory load compared to high-*K* individuals, display SPCN amplitude increments both when presented with relevant and irrelevant information (e.g., Vogel et al., 2005). This strongly suggests that visual working memory capacity is strictly intertwined with selective attention, in that, contrary to low-*K* individuals, high-*K* individuals are endowed not only with more capacity, but also with more efficient attention control for selecting the type of (relevant) information that is ultimately stored in visual working memory. This fact is all the more important when seeking to make sense of the absence of a correlation between visual working memory and numerical acuity. If we, like others, hypothesize that an approximate sense for numerosity is a sensory primitive in perception, much like, e.g., color, orientation, and motion, then numerosity is a signal that is embedded in early forward volleys of visual information flowing from posterior to anterior areas and, as such, is more than likely processed pre-attentively and unselectively (cfr., Lamme and Roelfsema, 2000). More work will be required to test this specific hypothesis, based on the rationale that numerical acuity should not vary as a function of the availability of attentional resources. One prediction that future work should address is that numerical acuity ought not to be impacted by an attentional blink (Raymond et al., 1992; see also Dell’Acqua et al., 2012) when a dot-comparison array is displayed as the second target trailing a first target at short intervals in rapid serial visual presentation. This demonstration would make a nice tie with analogous demonstrations provided for other, putatively sensory-driven, dimensions (some reviewed by Cohen et al., 2012). Even more critically, numerical acuity could be estimated in a dot-comparison task implemented as the second task of a psychological refractory period design (PRP; Pashler, 1994), and proved to be unaffected by task overlap, in analogy with demonstrations provided for different, pre-attentively processed, sensory information (e.g., Lien et al., 2024).

If numerical acuity and visual working memory are separate systems, then they are also likely implemented in distinct neuronal substrates in the adult brain, and this may seem at odds with findings pertaining to the anatomical and functional organization of the brain processes engaged by these abilities. Neuroimaging studies exploring the hemodynamic correlates in change-detections tasks have long pointed to posterior regions of the dorsal pathway, the intra-parietal sulcus (IPS) in particular, as a core hub in the working memory maintenance of visual input. Relative to other areas along the fronto-parietal, or dorsal, pathway, whose activity increases linearly even for set-sizes larger than maximum *K*, activity in IPS increases up to set-sizes equal to maximum *K*, and plateaus thereafter. Together with analogous results of EEG explorations of posterior activity in change detection tasks (e.g., Vogel and Machizawa, 2004), this pattern of IPS activity has been taken as a hallmark of the involvement of IPS in the encoding and maintenance of visual working memory representations (Todd and Marois, 2004, 2005; Xu and Chun, 2006; Robitaille et al., 2010; Cowan et al., 2011; Brigadoi et al., 2017). Investigations of the hemodynamic correlates of numerosity processing have indicated IPS as a core hub also for numerical cognition (Piazza et al., 2004, 2007; Arsalidou and Taylor, 2011). More specifically, IPS neurons have been shown to be organized in topographical maps of numerosities (Harvey et al., 2013). This neuroanatomical overlap of the cortical regions involved in visual working memory and numerosity processing may subtend a common functional scaffolding for representing both numbers and visual input in working memory. The dorsal pathway is primarily responsible for the processing of spatial information by the adult brain, likely for visuo-motor mapping operations subserving action (Milner and Goodale, 1995). Of import, the semantics of numbers has been hypothesized to be inherently spatial (e.g., Nieder and Dehaene, 2009; Cutini et al., 2014; Leibovich et al., 2017; Basso Moro et al., 2018, for a review) and information that is ultimately necessary for the generation of visual working memory traces is also spatial (e.g., Courtney et al., 1996; Harrison et al., 2010; Pertzov and Husain, 2014; Cai et al., 2019).

The present results can however be reconciled with the hypothesis of distinct neural substrates for visual working memory and numerical acuity by referring to a particularly recent set of findings suggesting that IPS is not the sole cortical region populated by neurons tuned to numerosity. Kutter et al. (2018) recorded activity from neurons in the mid-temporal cortex of neurosurgical patients involved in a calculation task requiring addition or subtraction of quantities expressed symbolically (i.e., digits) and non-symbolically (i.e., arrays of dots) and showed selective tuning of these neurons to specific numbers in either format. The population of neurons tuned selectively to numerical values expressed as digits were largely segregated from the population of neurons tuned selectively to numerical values expressed as arrays of dots. That is, neurons in the bilateral mid-temporal lobe coded either the numerosity of arrays of dots or the semantics of digits, but not both. Neurons with properties akin to IPS neurons have been described by Cai et al. (2023) in a 7-T fMRI exploration using a paradigm similar to that used by Kutter et al. (2018). Like IPS neurons, neurons organized in topographic maps tuned to numbers expressed both symbolically and non-symbolically have been found in the temporal-occipital cortex. Via the parahippocampal cortex, activity in these cortical areas is likely to provide numerosity information to IPS for the generation of semantic codes for numbers (Kutter et al., 2018). The widespread involvement of large portions of the posterior visual cortex up to and including frontal regions seems to be the rule rather than the exception, even when numbers must be processed in tasks like subitizing and visual exact enumerations (Demeyere et al., 2012), with some of these areas showing an interesting overlap with areas controlling visuo-spatial attention shifts (Sathian et al., 1999). In general, therefore, IPS may be a cortical region that two particularly extended fronto-parietal networks, one subserving visual working memory (e.g., Ungerleider et al., 1998), the other number perception (e.g., Nieder and Dehaene, 2009), share, without, however, necessarily implying any form of informational cross-talk between them. These anatomical considerations invite a final note of caution with reference to the generalizability of the present results to dot-comparison tasks using different numerosities than those used in the present study. In the present dot-comparison task, participants were presented with displays including 12–40 dots. A patient recently described by Anobile et al. (2020), who suffered an extended cortical insult as a result of hypoxic brain injury, displayed a performance in a dot-comparison task that was comparable to that of controls for numerosities of 12–16 dots, and a dramatically impaired performance for both smaller (i.e., in the subitizing range) and larger (64–128) numerosities. Anobile et al. (2020) have taken this neuropsychological pattern to reflect the existence of three distinct systems uniquely tuned to distinct numerosity ranges, one of which (i.e., the intermediate system) appears to be specifically involved in processing dot numerosities that coincide with those classically tested in neurologically intact participants, as we did in the present context. At the most general level, this indicates that the ability to process numerical quantities is unlikely to be a monolithic construct, raising possible questions about the specific aspect of number processing that appears to be dissociated from visual working memory (e.g., Anobile et al., 2019). More specifically, however, a single case report may not be perhaps a sufficiently solid empirical ground to question the generalizability of the present results. It is clear that this note of caution ought to be taken into serious consideration, should reports of cases like that described by Anobile et al. (2020) increase following their seminal observation.

In conclusion, our study provides a refined and reliable examination of the relationship between working memory capacity and numerical acuity. The independence observed between these cognitive processes — suggested by the absence of a correlation between the specific psychophysical measures used in the present empirical investigation — deepens our understanding of the boundaries and interactions of working memory within the broader cognitive system.

## Data availability statement

The raw data supporting the conclusions of this article are available at https://osf.io/qv8e5.

## Ethics statement

The studies involving humans were approved by comitato.etico.area17@unipd.it. The studies were conducted in accordance with the local legislation and institutional requirements. The participants provided their written informed consent to participate in this study.

## Author contributions

RD’A: Conceptualization, Funding acquisition, Investigation, Methodology, Resources, Supervision, Writing – original draft. PS: Conceptualization, Investigation, Methodology, Supervision, Writing – original draft. SB: Conceptualization, Data curation, Methodology, Writing – original draft. JG: Conceptualization, Investigation, Methodology, Supervision, Writing – original draft. RL: Conceptualization, Data curation, Investigation, Methodology, Writing – original draft. MD: Data curation, Formal analysis, Writing – original draft.

## References

[ref1] Anderson J. R. (2007). How can the human mind occur in the physical universe? New York, NY: Oxford University Press.

[ref2] Anobile G. Arrighi R. Burr D. C. (2019). Simultaneous and sequential subitizing are separate systems, and neither predicts math abilities. J. Exp. Child Psychol. 178, 86–103. doi: 10.1016/j.jecp.2018.09.017, PMID: 30380457

[ref3] Anobile G. Tomaiuolo F. Campana S. Cicchini G. M. (2020). Three-systems for visual numerosity: a single case study. Neuropsychologia 136:107259. doi: 10.1016/j.neuropsychologia.2019.107259, PMID: 31726066

[ref4] Arsalidou M. Taylor M. J. (2011). Is 2+2=4? Meta-analyses of brain areas needed for numbers and calculations. NeuroImage 54, 2382–2393. doi: 10.1016/j.neuroimage.2010.10.009 20946958

[ref5] Balaban H. Fukuda K. Luria R. (2019). What can half a million change detection trials tell us about visual working memory? Cognition 191:103984. doi: 10.1016/j.cognition.2019.05.021, PMID: 31234117

[ref6] Barth H. Kanwisher N. Spelke E. (2003). The construction of large number representations in adults. Cognition 86, 201–221. doi: 10.1016/S0010-0277(02)00178-6 12485738

[ref7] Basso Moro S. Dell’Acqua R. Cutini S. (2018). The SNARC effect is not a unitary phenomenon. Psychon. Bull. Rev. 25, 688–695. doi: 10.3758/s13423-017-1408-3 29264847

[ref8] Bays P. M. Husain M. (2008). Dynamic shifts of limited working memory resources in human vision. Science 321, 851–854. doi: 10.1126/science.1158023, PMID: 18687968 PMC2532743

[ref9] Becker M. Klößner S. (2023). PearsonDS: Pearson distribution system. R package version 1.2.4. Available at: https://CRAN.R-project.org/package=PearsonDS

[ref10] Benjamini Y. Hochberg Y. (1995). Controlling the false discovery rate: a practical and powerful approach to multiple testing. J. Royal Statistical Soc. 57, 289–300. doi: 10.1111/j.2517-6161.1995.tb02031.x

[ref11] Brigadoi S. Cutini S. Meconi F. Castellaro M. Sessa P. Marangon M.. (2017). On the role of the inferior intraparietal sulcus in visual working memory for lateralized single-feature objects. J. Cogn. Neurosci. 29, 337–351. doi: 10.1162/jocn_a_01042, PMID: 27626222

[ref12] Burr D. Ross J. (2008). A visual sense of number. Curr. Biol. 18, 425–428. doi: 10.1016/j.cub.2008.02.052 18342507

[ref13] Burr D. C. Turi M. Anobile G. (2010). Subitizing but not estimation of numerosity requires attentional resources. J. Vis. 10:20. doi: 10.1167/10.6.20 20884569

[ref14] Cai Y. Hofstetter S. Dumoulin S. O. (2023). Nonsymbolic numerosity maps at the occipitotemporal cortex respond to symbolic numbers. J. Neurosci. 43, 2950–2959. doi: 10.1523/JNEUROSCI.0687-22.2023, PMID: 36922026 PMC10124950

[ref15] Cai Y. Sheldon A. D. Yu Q. Postle B. R. (2019). Overlapping and distinct contributions of stimulus location and of spatial context to nonspatial visual short-term memory. J. Neurophysiol. 121, 1222–1231. doi: 10.1152/jn.00062.2019, PMID: 30856041 PMC6485733

[ref16] Cohen M. A. Cavanagh P. Chun M. M. Nakayama K. (2012). The attentional requirements of consciousness. Trends Cogn. Sci. 16, 411–417. doi: 10.1016/j.tics.2012.06.013 22795561

[ref17] Courtney S. M. Ungerleider L. G. Keil K. Haxby J. V. (1996). Object and spatial visual working memory activate separate neural systems in human cortex. Cereb. Cortex 6, 39–49. doi: 10.1093/cercor/6.1.39, PMID: 8670637

[ref18] Cowan N. (2001). The magical number 4 in short-term memory: a reconsideration of mental storage capacity. Behav. Brain Sci. 24, 87–114. doi: 10.1017/S0140525X01003922, PMID: 11515286

[ref19] Cowan N. Elliott E. M. Saults S. J. Morey C. C. Mattox S. Hismjatullina A.. (2005). On the capacity of attention: its estimation and its role in working memory and cognitive aptitudes. Cogn. Psychol. 51, 42–100. doi: 10.1016/j.cogpsych.2004.12.001, PMID: 16039935 PMC2673732

[ref20] Cowan N. Li D. Moffitt A. Becker T. M. Martin E. A. Saults J. S.. (2011). A neural region of abstract working memory. J. Cogn. Neurosci. 23, 2852–2863. doi: 10.1162/jocn.2011.21625, PMID: 21261453 PMC3138911

[ref21] Cutini S. Scarpa F. Scatturin P. Dell’Acqua R. Zorzi M. (2014). Number-space interactions in the human parietal cortex: enlightening the SNARC effect with functional near-infrared spectroscopy. Cereb. Cortex 24, 444–451. doi: 10.1093/cercor/bhs321, PMID: 23081883

[ref22] De Marco D. Cutini S. (2020). Introducing CUSTOM: a customized, ultraprecise, standardization-oriented, multipurpose algorithm for generating nonsymbolic number stimuli. Behav. Res. Methods 52, 1528–1537. doi: 10.3758/s13428-019-01332-z, PMID: 31965476

[ref23] Dehaene S. (1993). “Symbols and quantities in parietal cortex: elements of a mathematical theory of number representation and manipulation” in Attention and performance XXII: Sensorimotor foundations of higher cognition. eds. Haggard P.. (Oxford, UK: Oxford Academic), 527–574.

[ref24] Dehaene S. (2011). The number sense: How the mind creates mathematics. New York, NY: Oxford University Press.

[ref25] Dehaene S. Kerszberg M. Changeux J. P. (1998). A neuronal model of a global workspace in effortful cognitive tasks. Proceed. Natl. Acad. Sci. 95, 14529–14534. doi: 10.1073/pnas.95.24.14529, PMID: 9826734 PMC24407

[ref26] Dell’Acqua R. Dux P. E. Wyble B. Jolicœur P. (2012). Sparing from the attentional blink is not spared from structural limitations. Psychon. Bull. Rev. 19, 232–238. doi: 10.3758/s13423-011-0209-3 22215469

[ref27] Demeyere N. Rotshtein P. Humphreys G. W. (2012). The neuroanatomy of visual enumeration: differentiating necessary neural correlates for subitizing versus counting in a neuropsychological voxel-based morphometry study. J. Cogn. Neurosci. 24, 948–964. doi: 10.1162/jocn_a_00188, PMID: 22220729

[ref28] Drew T. Vogel E. K. (2008). Neural measures of individual differences in selecting and tracking multiple moving objects. J. Neurosci. 28, 4183–4191. doi: 10.1523/JNEUROSCI.0556-08.2008, PMID: 18417697 PMC2570324

[ref29] Ester E. F. Drew T. Klee D. Vogel E. K. Awh E. (2012). Neural measures reveal a fixed item limit in subitizing. J. Neurosci. 32, 7169–7177. doi: 10.1523/JNEUROSCI.1218-12.2012, PMID: 22623661 PMC3370889

[ref30] Fukuda K. Woodman G. F. (2017). Visual working memory buffers information retrieved from visual long-term memory. Proceed. Natl. Acad. Sci. 114, 5306–5311. doi: 10.1073/pnas.1617874114, PMID: 28461479 PMC5441785

[ref31] Fukuda K. Woodman G. F. Vogel E. K. (2015). “Individual differences in visual working memory capacity: contributions of attentional control to storage” in Attention and performance XXV: mechanisms of sensory working memory. eds. Jolicœur P. Lefebvre C. Martinez-Trujillo J. (New York, NY: Academic Press), 105–119.

[ref32] Gallistel C. R. Gelman R. (1992). Preverbal and verbal counting and computation. Cognition 44, 43–74. doi: 10.1016/0010-0277(92)90050-R 1511586

[ref33] Gaspar J. M. Christie G. J. Prime D. J. Jolicœur P. McDonald J. J. (2016). Inability to suppress salient distractors predicts low visual working memory capacity. Proceed. Natl. Acad. Sci. 113, 3693–3698. doi: 10.1073/pnas.1523471113, PMID: 26903654 PMC4822617

[ref34] Gennari G. Dehaene S. Valera C. Dehaene-Lambertz G. (2023). Spontaneous supra-modal encoding of number in the infant brain. Curr. Biol. 33, 1906–1915.e6. doi: 10.1016/j.cub.2023.03.062, PMID: 37071994

[ref35] Green D. M. Swets J. A. (1974). Signal detection theory and psychophysics. Huntington, NY: Krieger.

[ref36] Harrison A. Jolicœur P. Marois R. (2010). “What” and “where” in the intraparietal sulcus: an fMRI study of object identity and location in visual short-term memory. Cereb. Cortex 20, 2478–2485. doi: 10.1093/cercor/bhp314, PMID: 20100899 PMC2936801

[ref37] Harvey B. M. Klein B. P. Petridou N. Dumoulin S. O. (2013). Topographic representation of numerosity in the human parietal cortex. Science 341, 1123–1126. doi: 10.1126/science.1239052 24009396

[ref38] Jeffreys H. (1939). Theory of probability (III Ed.). New York, NY: Oxford University Press.

[ref39] Johnson M. K. McMahon R. P. Robinson B. M. Harvey A. N. Hahn B. Leonard C. J.. (2013). The relationship between working memory capacity and broad measures of cognitive ability in healthy adults and people with schizophrenia. Neuropsychology 27, 220–229. doi: 10.1037/a0032060, PMID: 23527650 PMC3746349

[ref40] Jolicœur P. Dell’Acqua R. (1998). The demonstration of short-term consolidation. Cogn. Psychol. 36, 138–202. doi: 10.1006/cogp.1998.0684, PMID: 9721199

[ref41] Kutter E. F. Bostroem J. Elger C. E. Mormann F. Nieder A. (2018). Single neurons in the human brain encode numbers. Neuron 100, 753–761.e4. doi: 10.1016/j.neuron.2018.08.036 30244883

[ref42] Lamme V. A. F. Roelfsema P. R. (2000). The distinct modes of vision offered by feedforward and recurrent processing. Trends Neurosci. 23, 571–579. doi: 10.1016/S0166-2236(00)01657-X, PMID: 11074267

[ref43] Lefebvre C. Dell’Acqua R. Roelfsema P. R. Jolicœur P. (2011). Surfing the attentional waves during visual curve tracing: evidence from the sustained posterior contralateral negativity. Psychophysiology 48, 1510–1516. doi: 10.1111/j.1469-8986.2011.01228.x, PMID: 21770971

[ref44] Leibovich T. Katzin N. Harel M. Henik A. (2017). From “sense of number” to “sense of magnitude”: the role of continuous magnitudes in numerical cognition. Behav. Brain Sci. 40:e164. doi: 10.1017/S0140525X16000960, PMID: 27530053

[ref45] Lien M.-C. Ruthruff E. Tolomeo D. (2024). Evidence that proactive distractor suppression does not require attentional resources. Psychon. Bull. Rev. doi: 10.3758/s13423-023-02422-y 38049572

[ref46] Luck S. J. Vogel E. K. (2013). Visual working memory capacity: from psychophysics and neurobiology to individual differences. Trends Cogn. Sci. 17, 391–400. doi: 10.1016/j.tics.2013.06.006, PMID: 23850263 PMC3729738

[ref47] Luria R. Sessa P. Gotler A. Jolicœur P. Dell’Acqua R. (2010). Visual short-term memory capacity for simple and complex objects. J. Cogn. Neurosci. 22, 496–512. doi: 10.1162/jocn.2009.21214, PMID: 19301998

[ref48] Ly A. Verhagen J. Wagenmakers E. J. (2016). Harold Jeffreys’s default Bayes factor hypothesis tests: explanation, extension, and application in psychology. J. Math. Psychol. 72, 19–32. doi: 10.1016/j.jmp.2015.06.004

[ref49] Meyer D. E. Kieras D. E. (1997). A computational theory of executive cognitive processes and human multiple-task performance: part 1 basic mechanisms. Psychol. Rev. 104, 3–65. doi: 10.1037/0033-295x.104.1.3, PMID: 9009880

[ref50] Milner A. D. Goodale M. A. (1995). The visual brain in action. Oxford, UK: Oxford University Press.

[ref51] Miyake A. Friedman N. P. Rettinger D. A. Shah P. Hegarty M. (2001). How are visuospatial working memory, executive functioning, and spatial abilities related? A latent-variable analysis. J. Exp. Psychol. Gen. 130, 621–640. doi: 10.1037/0096-3445.130.4.621, PMID: 11757872

[ref52] Nieder A. Dehaene S. (2009). Representation of number in the brain. Annu. Rev. Neurosci. 32, 185–208. doi: 10.1146/annurev.neuro.051508.135550 19400715

[ref53] Pashler H. (1988). Familiarity and visual change detection. Percept. Psychophys. 44, 369–378. doi: 10.3758/BF03210419 3226885

[ref54] Pashler H. (1994). Dual-task interference in simple tasks: data and theory. Psychol. Bull. 116, 220–244. doi: 10.1037/0033-2909.116.2.220, PMID: 7972591

[ref55] Pertzov Y. Husain M. (2014). The privileged role of location in visual working memory. Atten. Percept. Psychophys. 76, 1914–1924. doi: 10.3758/s13414-013-0541-y, PMID: 24027033 PMC4212176

[ref56] Piazza M. Fumarola A. Chinello A. Melcher D. (2011). Subitizing reflects visuo-spatial object individuation capacity. Cognition 121, 147–153. doi: 10.1016/j.cognition.2011.05.007, PMID: 21679934

[ref57] Piazza M. Izard V. Pinel P. Le Bihan D. Dehaene S. (2004). Tuning curves for approximate numerosity in the human intraparietal sulcus. Neuron 44, 547–555. doi: 10.1016/j.neuron.2004.10.014, PMID: 15504333

[ref58] Piazza M. Pinel P. Le Bihan D. Dehaene S. (2007). A magnitude code common to numerosities and number symbols in human intraparietal cortex. Neuron 53, 293–305. doi: 10.1016/j.neuron.2006.11.022, PMID: 17224409

[ref59] Pratte M. S. Green M. L. (2023). Systematic differences in visual working memory performance are not caused by differences in working memory storage. J. Exp. Psychol. Learn. Mem. Cogn. 49, 335–349. doi: 10.1037/xlm0001202, PMID: 36729486 PMC10141665

[ref60] Ramaty A. Luria R. (2018). Visual working memory cannot trade quantity for quality. Front. Psychol. 9:719. doi: 10.3389/fpsyg.2018.00719, PMID: 29881361 PMC5976751

[ref61] Raymond J. E. Shapiro K. L. Arnell K. M. (1992). Temporary suppression of visual processing in an RSVP task: an attentional blink? J. Exp. Psychol. Hum. Percept. Perform. 18, 849–860. doi: 10.1037/0096-1523.18.3.849, PMID: 1500880

[ref62] Revkin S. K. Piazza M. Izard V. Cohen L. Dehaene S. (2008). Does subitizing reflect numerical estimation? Psychol. Sci. 19, 607–614. doi: 10.1111/j.1467-9280.2008.02130.x, PMID: 18578852

[ref63] Robitaille N. Marois R. Todd J. J. Grimault S. Cheyne D. Jolicœur P. (2010). Distinguishing between lateralized and nonlateralized brain activity associated with visual short-term memory: fMRI, MEG, and EEG evidence from the same observers. NeuroImage 53, 1334–1345. doi: 10.1016/j.neuroimage.2010.07.027, PMID: 20643214

[ref64] Rouder J. N. Morey R. D. Morey C. C. Cowan N. (2011). How to measure working memory capacity in the change detection paradigm. Psychon. Bull. Rev. 18, 324–330. doi: 10.3758/s13423-011-0055-3, PMID: 21331668 PMC3070885

[ref65] Sathian K. Simon T. J. Peterson S. Patel G. A. Hoffman J. M. Grafton S. T. (1999). Neural evidence linking visual object enumeration and attention. J. Cogn. Neurosci. 11, 36–51. doi: 10.1162/089892999563238, PMID: 9950713

[ref66] Todd J. J. Marois R. (2004). Capacity limit of visual short-term memory in human posterior parietal cortex. Nature 428, 751–754. doi: 10.1038/nature02466 15085133

[ref67] Todd J. J. Marois R. (2005). Posterior parietal cortex activity predicts individual differences in visual short-term memory capacity. Cognit. Affect. Behav. Neurosci. 5, 144–155. doi: 10.3758/CABN.5.2.144, PMID: 16180621

[ref68] Trick L. M. Pylyshyn Z. W. (1994). Why are small and large numbers enumerated differently? A limited-capacity preattentive stage in vision. Psychol. Rev. 101, 80–102. doi: 10.1037/0033-295X.101.1.80, PMID: 8121961

[ref69] Ungerleider L. G. Courtney S. M. Haxby J. V. (1998). A neural system for human visual working memory. Proceed. Natl. Acad. Sci. 95, 883–890. doi: 10.1073/pnas.95.3.883, PMID: 9448255 PMC33812

[ref70] Unsworth N. Fukuda K. Awh E. Vogel E. K. (2014). Working memory and fluid intelligence: capacity, attention control, and secondary memory retrieval. Cogn. Psychol. 71, 1–26. doi: 10.1016/j.cogpsych.2014.01.003, PMID: 24531497 PMC4484859

[ref71] Unsworth N. Fukuda K. Awh E. Vogel E. K. (2015). Working memory delay activity predicts individual differences in cognitive abilities. J. Cogn. Neurosci. 27, 853–865. doi: 10.1162/jocn_a_00765, PMID: 25436671 PMC4465247

[ref72] Vogel E. K. Machizawa M. G. (2004). Neural activity predicts individual differences in visual working memory capacity. Nature 428, 748–751. doi: 10.1038/nature02447, PMID: 15085132

[ref73] Vogel E. K. McCollough A. W. Machizawa M. G. (2005). Neural measures reveal individual differences in controlling access to working memory. Nature 438, 500–503. doi: 10.1038/nature04171, PMID: 16306992

[ref74] Xu Y. Chun M. M. (2006). Dissociable neural mechanisms supporting visual short-term memory for objects. Nature 440, 91–95. doi: 10.1038/nature04262, PMID: 16382240

[ref75] Zhang W. Luck S. J. (2008). Discrete fixed-resolution representations in visual working memory. Nature 453, 233–235. doi: 10.1038/nature06860, PMID: 18385672 PMC2588137

